# Comparative analysis of prescription patterns and errors in government versus private hospitals in Dhaka: A cross‐sectional study

**DOI:** 10.1002/hsr2.2302

**Published:** 2024-08-12

**Authors:** Md Abdus Samad, K. M. Yasif Kayes Sikdar, Ashfia Tasnim Munia, Farhan Tanvir Patwary, Md Raihan Sarkar, Md. Rashidul Islam Rashed

**Affiliations:** ^1^ Department of Pharmacy, Faculty of Pharmacy University of Dhaka Dhaka Bangladesh; ^2^ Department of Pharmacy, School of Pharmaceutical Sciences State University of Bangladesh Dhaka Bangladesh; ^3^ Department of Pharmaceutical Technology, Faculty of Pharmacy University of Dhaka Dhaka Bangladesh; ^4^ Institute of Statistical Research and Training University of Dhaka Dhaka Bangladesh; ^5^ Department of Pharmacy University of Asia Pacific Dhaka Bangladesh

**Keywords:** inscriptions, polypharmacy, Prescription patterns and errors, subscriptions, superscriptions

## Abstract

**Background and Objectives:**

Prescription errors can inadvertently compromise the effectiveness and increase the risk of adverse events. This study aims to compare prescription patterns and errors between government and private hospitals in Dhaka, Bangladesh, by evaluating the World Health Organization (WHO) prescription indicators, polypharmacy, and omission errors.

**Methods:**

Between September 2021 and November 2021, a total of 399 prescriptions were collected from outpatient departments of various government and private hospitals from patients or their attendants. The data were analyzed using the statistical package STATA 15. Chi‐square and Fisher's exact test were employed to determine associations (*p* < 0.05) among various types of categorical data.

**Results:**

Of the collected prescriptions, 48% (*n* = 192) were from government, while 52% (*n* = 207) were from private hospitals. The mean number of medicines per prescription was 5.16 for government and 5.87 for private hospitals. Generic names were absent (0%) in both types of hospitals. Antibiotics were present in 34.37% of prescriptions from government and 51.69% from private hospitals. Moreover, injection were found in 17.70% of government and 18.35% of private hospitals' prescriptions. Government hospitals adhered to 67.97% of the essential drug list, whereas private hospitals adhered to 80.42%. Associations between hospital types were observed in missing age, and comorbidities, while no association was found in inscription mistakes. Missing dates and signatures were also associated with hospital types. Polypharmacy was observed in 49.47% of government hospitals and 71.01% of private hospitals. Additionally, polypharmacy in females, pediatrics, geriatrics, and missing comorbidity were also associated with hospital types (*p* < 0.05).

**Conclusion:**

Both government and private hospitals exhibited similar deviations from the WHO prescribing indicators. While government hospitals showed more omission errors, private hospitals exhibited higher rates of polypharmacy. Physicians in both types of hospitals should be vigilant about omission errors, maintain the WHO prescribing indicators, and minimize polypharmacy.

## 1 INTRODUCTION

The ideal prescription aims to heighten effectiveness, minimize risks and costs (by prescribing lower‐cost generic medicines instead of patent drugs whenever possible), and maintain the steadiness of risks and benefits. Additionally, it esteems the patient's right to be involved in their treatment decisions.^1^ However, any wrong prescription writing process or prescriber decision which can inadvertently reduces the quality and effectiveness of the treatment or augments the risk of the adverse event is defined as prescription error.^2^ These can occur at any step during prescription writing.^3^ Slips, lapses, omissions in drug writing, failure to select appropriate doses, mistakes in transcribing medications, and illegible handwriting are responsible for such errors.^3^ They are accounted for 70% of medication errors.^3^ These medication errors augment adverse effects, drug dependency on patients, morbidity and mortality rate, the risk of resistance to antimicrobials, and overall demand for the drug.^4^ Ultimately, medication errors diminish patient confidence and contribute to a growing distrust in the healthcare system.^5^

The World Health Organization (WHO) reported that half of medications are prescribed, sold, or dispensed improperly.^4^ Additionally, each year, between 7000 and 9000 individuals lose their lives due to medication errors. Hundreds of thousands more suffer from adverse reactions or other medication‐related complications but do not formally report them.^5^ Globally, medication errors carry an estimated annual economic burden of $42 billion.^6^ In light of these alarming statistics, the WHO launched the “Medication without Harm” initiative in March 2017. The primary objective of this initiative is to garner increased global attention and to halve the incidence of severe, preventable harm associated with medications across all countries within the next 5 years.^6^

Some studies have been done in previous times in Bangladesh. In 2015, a study revealed: “the absence of drug strength, no dose adjustment of renal and hepatic patients, serious drugs‐drugs interaction, and medication‐related problems” that increased treatment costs and adverse event rates.^7^ In 2016, another study showed a myriad number of the absence of gender, age, patient's name, drug information, dosage regimens, dosage forms, prescriber's sign, and prescription date.^8^

The main objective of this research was to analyze and compare prescription errors and patterns between government and private hospitals in Dhaka, Bangladesh. Where WHO prescribing indicators were used as a benchmark.^9^ The study investigates various types of errors including omissions such as inscriptions, superscriptions, subscriptions, and instances of polypharmacy within both types of hospital settings. Additionally, the errors and trends were compared with those of some South Asian countries to illustrate the differences in prescription patterns and errors between private and government hospitals and neighboring countries. Though some studies in Bangladesh illustrate the various prescription errors, as per our knowledge, studies have yet to conduct such a comparison and relationship of prescription errors within Dhaka city. The investigation could assist healthcare authorities of both sectors in Dhaka city. This study may be supportive of finding out the current status of prescriptions in both types of hospitals. It also assists in establishing a unique framework of prescriptions in Dhaka City, Bangladesh, that ultimately improves the overall healthcare quality of Bangladesh.

## 2 METHOD AND MATERIALS

### 2.1 Study design

From November 2021 to June 2022, a cross‐sectional investigation to evaluate the difference of prescription errors among the government and private hospitals was conducted at outdoor unit of fifteen different specialized and general tertiary‐level hospitals in Dhaka, Bangladesh. Moreover, this cross‐sectional analysis was carried out following the guidelines of Strengthening the Reporting of Observational Studies in Epidemiology (STROBE).^10^

### 2.2 Sample size determination

The sample size was calculated by Rasoft®, Inc. The population in Dhaka city was predicted to be more than the hundreds of thousands, about 230 million people.^11^ As a result, the least expected number of participants was calculated to be 384, with a 95% confidence level. Nearly 4% population were considered as nonresponsive participants. So, the overall sample size of this study was 399.

This sample size was distributed across hospitals using the “n^th^” random sampling technique. A proportionate distribution method was employed to select participants from each hospital. Specifically, the formula *p* = n*(*S*/*T*), where *p* represents the sample size from a particular hospital, *n* is the total calculated sample size, *T* = the total number of beds in the selected hospitals, *S* = the number of beds in a particular hospital.^12^ Among the randomly collected 399 prescriptions, 192 prescriptions were from government hospitals, while the remaining 207 were from private hospitals.

### 2.3 Sampling method and technique

This investigation utilized a two‐step sampling approach. Initially, a selection process was undertaken to identify a number of government and private hospitals for data collection. Following the hospital selection, participants were chosen from these hospitals until the calculated sample size was achieved.

#### 2.3.1 Selecting hospitals for the investigations

The selection of hospitals for the investigation involved considering only tertiary‐level hospitals in Dhaka city, with other types being excluded. Utilizing information from the Bangladeshi government database,^13^, ^14^ registered tertiary private and government hospitals were listed, and their bed numbers were recorded based on information provided by hospital authorities. Subsequently, the “*n*th” sampling technique (*n* = 2nd) was employed to select the final hospitals for data collection. Ultimately, 15 hospitals were chosen for data collection from patients (Supporting Information S1: Table 1).

#### 2.3.2 Participants' selections from the selected hospitals

The respondents were included in the investigation using consecutive sampling techniques, wherein all eligible individuals from the respective hospitals were continuously included until the predetermined sample size of selected hospitals was achieved.

### 2.4 Data collection and inclusion criteria

Undergraduate pharmacy students, upon fulfilling their instruction, were enrolled as data collectors. Data collection included getting consent via a form and capturing participants' prescriptions obtained postphysician visits. Moreover, data were collected on a one‐prescription‐per‐patient system to assure accuracy and avoid duplication. Patients of various ages and genders were considered for the investigation. To ensure the presence of a wide range of ailment and various types of patients, no any types of disease and patients get priority. Children, severe patients, and pregnant women who were unable to write but can speak were included with permission from their caretakers. However, individuals who were unable to talk or were unconscious throughout the inquiry were eliminated.

### 2.5 Study measures and definition

#### 2.5.1 WHO prescribing indicators

The WHO prescribing indicators were accounted as standard to calculate the divergence of prescription patterns between private and government hospitals.^9^ These indicators included:

- Average number of drugs prescribed per encounter (optimal value: 1.6–1.8).
- Percentage of drugs prescribed by generic name (optimal value: 100%).
- Percentage of encounters where an antibiotic was prescribed (optimal value: 20.0–26.8%).
- Percentage of encounters with at least one injection prescribed (optimal value: 13.4–24.1%).
- Percentage of drugs prescribed from the WHO Essential Drugs List (EDL) (optimal value: 100%).

#### 2.5.2 Various types of errors

Various types of omission errors, including subscriptions, inscriptions, and superscriptions, were analyzed to identify differences in prescription patterns. As classifications of these errors may vary across different sources of literature due to diverse interpretations, a precise criteria was established based on the study conducted by Shahaibi et al.^15^ to ensure consistency in assessment.

##### Superscription errors

The types of omission errors are classified based on the information omitted regarding patient age, weight, gender, and comorbidity.^15^

##### Inscription errors

The types of omission errors considered for inscriptions include incomplete information on dose frequency, dosage form, dose, and dose duration.^15^

##### Subscription errors

The types of omission errors classified as subscriptions include missing information on the date and prescriber's signature.^15^

#### 2.5.3 Polypharmacy

According to Viktil et al. (2007), polypharmacy is defined as the concomitant use of five or more drugs.^16^ Various categories of data, such as gender, age, comorbidity, and prescription errors (missing age and missing comorbidities), were considered to calculate the association of polypharmacy with hospital types.

### 2.6 Statistical analysis

All data were meticulously entered into Microsoft Excel 2016 for preservation and subsequent analysis. The analyses were conducted using the Software for Statistics and Data Science (STATA) 15 (StataCorp LLC). The frequency and percentage of various categorical data, such as prescription errors (omission of superscriptions, inscriptions, subscriptions) and polypharmacy (gender, age, comorbidity, and prescription errors), were calculated. Furthermore, age was categorized following the approach used in the study by Horng et al. because the classification of age groups in this current study was based on facial characteristics.^17^ During the analysis, collected sample of this investigation was disseminated into two groups such as government and private hospitals. Then all the data of various errors groups were categorized into two groups such as absence of errors (No) or presence of errors (Yes), while for polypharmacy consideration it was presence of polypharmacy (Yes) or absence of polypharmacy (No). All the nominal data was considered for the associations rather than the numeric data during the investigation. For this mutually exclusive categorical variables, Chi‐square statistic test was employed to calculate the associations.^18^, ^19^, ^20^ However, Fisher's exact test was implemented if the any categorical variable number of incident is less than 5. A statistical association was considered if the *p* was ≤ 0.05.

### 2.7 Ethical clearance

The investigation followed the Helsinki Declaration of the World Medical Association (WMA). However, neither humans nor animals were hurt; it was not part of any clinical trial. Moreover, each participant's verbal and/or written consent was obtained before collecting their prescription. For children and young adults, their consent of them was ensured by them and their corresponding guardians. The prescription data were collected for solely research purposes, and the privacy and confidentiality of the participants was strictly preserved. Additionally, written or verbal permission was taken from the hospital's authority. However, before launching the investigation, the ethical permission was taken from “Ethical Review Committee” of the Faculty of Biological Sciences, University of Dhaka, and the ethical approval reference number was 148/Bio. Scs (attached in Supplorting Information S1 files).

## 3 RESULTS

### 3.1 Characteristics of study participants

**Figure 1:**
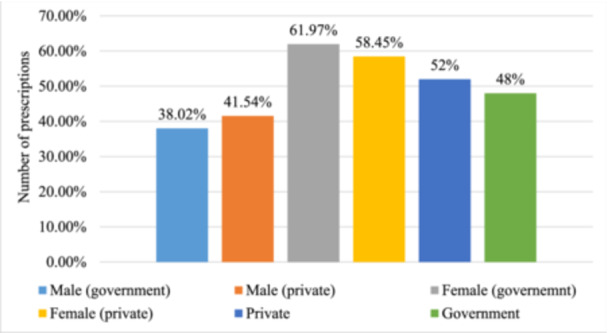
Number of prescriptions based on the gender and hospital types.

A total of 399 prescriptions were evaluated. Respectively, 207 (52%) and 192 (48%) prescriptions were collected from private and government hospitals in Dhaka city (Figure 1). Only gender and age sociodemographic criteria were considered. All variables were categorically coded Nearly 60% of both hospitals' participants were female (Figure 1).

**Figure 2:**
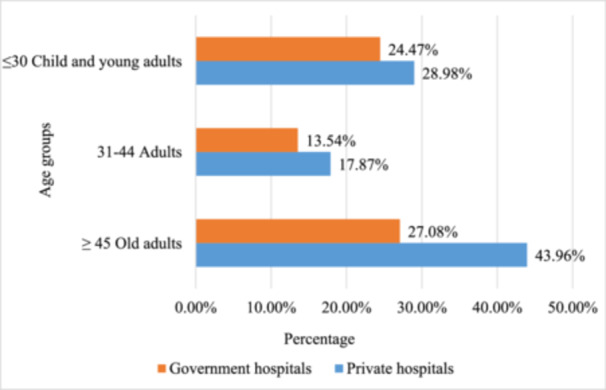
Number of prescriptions based on age.

From pediatrics to geriatrics, all age groups were considered, while age was categorized into several groups. The majority (43.96%) of the private participants were belonging to the old adults (aged ≥ 45) (Figure 2). However, a majority (34.89%) of the government hospitals had no patients' age (Table 3).

### 3.2 Medicines number in prescriptions

Government prescriptions exhibited the following medication counts: ≤1 (2.08%), 2 (8.33%), 3 (18.75%), 4 (21.35%), and ≥5 (49.47%). In contrast, private prescriptions displayed: ≤1 (0.48%), 2 (1.93%), 3 (7.24%), 4 (19.32%), and ≥5 (71.01%). As detailed in Table 1, our analysis underscores that government prescriptions tend to have a lower medication count compared to private prescriptions.

**Table 1:** Number of medicines per prescription (*n* = 399).

Number of medicines or drugs per prescription	Government Hospitals *n* = 192 (100%)	Private Hospitals *n* = 207 (100%)	Total number of prescriptions *n* = 399 (100%)
≤1	4 (2.08%)	1 (0.48%)	5 (1.25%)
2	16 (8.33%)	4 (1.93%)	20 (5.01%)
3	36 (18.75%)	15 (7.24%)	51 (12.78%)
4	41 (21.35%)	40 (19.32%)	81 (20.30%)
≥5	95 (49.47%)	147 (71.01%)	242 (60.65%)

### 3.3 Prescribing indicators

The “mean number of medications per prescription” for government and private hospitals was 5.16 and 5.87, respectively. Neither government nor private prescriptions included medications prescribed by their generic names, (0% percentage in both cases). Regarding the “percentage of prescriptions containing antibiotics,” government hospitals accounted for 34.37%, whereas private hospitals had a higher percentage at 51.69%. The “percentage of prescriptions featuring parenteral dosage forms” in government and private hospitals was 17.70% and 18.35%, respectively. Furthermore, regarding adherence to the EDL, government hospitals prescribed 67.97% medicine from the EDL, while private hospitals exceeded government hospitals (80.42% of the total medicine) (Table 2).

**Table 2:** WHO) prescribing indicators comparison in both government and private hospitals of Dhaka city (*n* = 399).

WHO prescription indicators	Acceptance range (Optimal value)	Government hospitals	Private hospitals	Comments
Average number of drugs per prescription	1.6−1.8	5.16	5.87	Out of range
Generic name of medicine or drugs	100%	0%	0%	Didn't contain any generic name
Percentage of antibiotic containing prescriptions	20%–26.8%	34.37% (*n* = 66)	51.69% (*n* = 107)	Out of range
Percentage of parenteral dosage form containing prescriptions	13.4–24.1%	17.70% (*n* = 34)	18.35% (*n* = 38)	Within range
Percentage of medicines from the WHO Essential Drugs List	100%	67.97% (*n* = 673)	80.42% (*n* = 978)	Behind of range

Abbreviation: WHO, World Health Organization.

### 3.4 Various prescribing omission errors

In terms of superscription errors, age and comorbidity were omitted more frequently in government hospitals (35.41% and 40.10%, respectively) compared to private hospitals (9.66% and 28.50%, respectively), with significant differences between hospital types (*p* < 0.05). Both types of hospitals omitted patients’ weight at nearly the same rate (86.45% and 88.88%, respectively), which was not statistically significant (*p* > 0.05) (Table 3). Additionally, 40.10% of government hospitals, compared to 28.50% of private hospitals, failed to mention comorbidities in their prescriptions (*p* < 0.05).

**Table 3:** Various prescribing omission errors of both government and private hospitals.

Errors		Government hospitals		Private hospitals		*p* Value
		(*n* = 192)		(*n* = 207)		
		Yes	No	Yes	No	
Superscription	Absence of age	67 (34.89%)	125 (65.11%)	19 (9.17%)	188 (90.82%)	<0.0001
	Absence of weight	166 (86.45%)	26 (13.54%)	184 (88.88%)	23 (11.11%)	>0.05
	Absence of comorbidity	77 (40.10%)	115 (59.9%)	59 (28.50%)	148 (71.5%)	<0.05
Inscription	Absence of dose frequency	21 (10.93%)	171 (89.06%)	14 (6.76%)	193 (93.23%)	>0.05
	Absence of dosage forms	12 (6.25%)	180 (93.75%)	7 (3.38%)	200 (96.61%)	>0.05
	Absence of dose	79 (41.14%)	113 (58.85%)	91 (43.96%)	116 (56.03%)	>0.05
	Absence of dose duration	40 (20.83%)	152 (79.16%)	35 (16.90%)	172 (83.09%)	>0.05
Subscription	Absence of date	11 (5.72%)	181 (94.28%)	2 (0.96%)	205 (99.04%)	<0.01
	Physician's signature	65 (33.85%)	127 (66.14%)	187 (90.33%)	20 (9.66%)	<0.0001

*Note*: *p* < 0.05 marked as significance. Association was calculated through Chi‐Square and Fisher exact testing.

Regarding inscription errors, the pattern of missing drug‐related information—such as dose frequency, dosage forms, dose, and dose duration—was similar in both types of hospitals (Table 3), and these errors were not significantly correlated with hospital type (*p* > 0.05). Among these errors, missing dose and dose duration were more prominent than others. In government hospitals, the rates were 41.14% and 20.83%, respectively, while in private hospitals, they were 43.96% and 16.90%, respectively (Table 3).

In terms of subscription errors, the absence of date and signature showed significant differences (*p* < 0.05) between hospital categories. Government hospitals had higher omission rates for date and signature (5.72% and 66.14%, respectively) compared to private hospitals (0.96% and 9.66%, respectively) (Table 3).

### 3.5 Polypharmacy

Socio‐demographic data of the participants was also considered to evaluate the polypharmacy scenario among the private and government hospitals. The data revealed that polypharmacy was present in approximately half (49.47%) of the government hospitals, whereas it was observed in more than two‐thirds (71.01%) of private hospitals' cases (Table 4).

**Table 4:** Determinants association of polypharmacy among government and private hospitals based on the various categories.

Variables		Polypharmacy (*n* = 242)		Nonpolypharmacy (*n* = 157)		*p* Value
		Government hospitals	Private hospitals	Government hospitals	Private hospitals	
Hospital's types		95 (49.47%)	147 (71.01%)	97 (50.52%)	60 (28.98%)	<0.0001
Gender	Male	42 (21.87%)	60 (28.98%)	31 (16.15%)	26 (12.56%)	>0.05
	Female	53 (27.60%)	87 (42.02%)	66 (34.37%)	34 (16.42%)	<0.0001
Categories of age	≤30 (pediatrics, young adults)	8 (4.17%)	40 (19.32%)	39 (20.31%)	20 (9.66%)	<0.0001
	31−44 (middle‐aged adults)	11 (5.72%)	24 (11.59%)	15 (7.81%)	13 (6.28%)	>0.05
	≥45 (old adults, geriatrics)	31 (16.14%)	71 (34.29%)	21 (10.93%)	20 (9.66%)	<0.05
Co‐morbidity	1	24 (12.5%)	50 (24.15%)	54 (28.125%)	35 (16.90%)	<0.001
	≥2	32 (16.67%)	55 (26.57%)	5 (2.60%)	8 (3.86%)	>0.05
Prescription's errors	Missing age	46 (23.95%)	11 (5.31%)	22 (11.45%)	9 (4.34%)	>0.05
	Missing comorbidity	39 (20.31%)	42 (20.28%)	38 (19.79%)	17 (8.21%)	>0.05

*Note*: *p* < 0.05 marked as significance. Association was calculated through Chi‐Square and Fisher exact testing.

In terms of gender categories, both types of hospitals had a nearly similar proportion of male participants, with no significant difference (*p* > 0.05). However, females with polypharmacy were more prevalent than males, particularly in private hospitals (34.37%), and this was significant (*p* < 0.05) (Table 4).

Among the age categories, polypharmacy was significantly more prevalent in pediatrics and young adults groups in private hospitals (20.31% vs. 4.17%, respectively; *p* < 0.001) (Table 4). For middle‐aged adults, the prevalence was similar in both types of hospitals (5.72% vs. 7.81%, respectively; *p* > 0.05). Although the frequency of polypharmacy cases was higher among geriatric participants in government hospitals (*n* = 31, 16.14%), the proportion revealed that polypharmacy in private hospitals was higher, with a 1:1 ratio (10.93% vs. 9.66%, polypharmacy vs. nonpolypharmacy, respectively), compared to the ratio in government hospitals (16.14% vs. 34.29%, or 0.47:1, polypharmacy vs. nonpolypharmacy, respectively) (*p* < 0.05) (Table 4).

Concerning co‐morbidity, a significant difference (*p* < 0.001) was observed in cases with a single co‐morbidity, with a higher percentage in private hospitals compared to government hospitals (28.12% vs. 12.5%) (Table 4). Conversely, incidents of multiple co‐morbidities with polypharmacy were more prevalent in government hospitals than in private hospitals, although the ratios were nearly equal (Table 4) (*p* > 0.05).

In the category of prescription errors involving missing co‐morbidity with polypharmacy, both government and private hospitals had a similar rate of incidents (20.31% vs. 19.79%, respectively). However, this difference was statistically significant (*p* < 0.05).

## 4 DISCUSSION

Prescription errors that ultimately lead to medication mistakes significantly reduce treatment quality and are considered a major health concern.^4^ For a better understanding of prescription errors and patterns across private and government hospitals in Dhaka, Bangladesh, a cross‐sectional study was needed based on the standard of WHO prescribing indicators and other omission errors such as superscriptions, inscriptions, subscriptions, and polypharmacy. The outcomes of this study revealed that both types of the hospital was far deviated than the WHO prescribing indicators. Though there was some variations, the number omission errors and prescribing more medicine especially polypharmacy were noticeable in both types of hospital. These prescription errors' may compromise the treatment goal overall the healthcare services.

### 4.1 Prescribing indicators

According to the findings, the “average number of medications per prescription” in government and private hospitals was 5.16 and 5.87, respectively. These results exceed the WHO recommendation, indicating that both types of hospitals prescribe a large amount of medication. In comparison, a review in India reported an average of 3.08 medications were suggested by per individual.^21^ A study in Nepal showed a mean frequency of 2.55 medicines.^22^ Another study conducted in Islamabad, Pakistan, revealed an average of 4.9 medicines prescribed per encounter.^23^ This comparison indicates that both government and private hospitals not only exceed the WHO recommendation but also prescribe more medications than other South Asian countries, particularly from India and Nepal. The high number of prescribed medications increases the frequency of prescription errors, which ultimately raises the risk of adverse effects such as drug‐drug interactions, side effects, patient noncompliance, and bacterial resistance to antimicrobial drugs.^24^

The generic name was missed in both government and private prescriptions in this investigation, while the WHO's optimal value was 100%. The Indian review data revealed that the percentage of using generic names in prescriptions could be varied from 0.05% to 97.7% based on the study region.^21^ An additional study performed in the capital of Pakistan, Islamabad, revealed that just 6.1% of medications were administered through their generic names.^23^ In another study done in Nepal, a significant majority of medications (57.5%), were administered under their generic names.^22^ Both government and private hospitals also deviated from the other studies of the south Asia. As a safety precaution, the WHO recommends using generic names because it allows for a greater exchange of information and better communication among healthcare providers.^25^ It will be a recommendation for the both types of hospitals' authority to provide especial emphasis on the usage of generic name.

The percentage of antibiotics prescribing in the government hospitals was higher (34.37%) than the WHO's optimal value, whereas the private was probably two times higher (51.69%). As per findings, one‐third of prescriptions of government hospitals as well as half of private hospitals have an infection case or prescribed excessive antibiotics in Dhaka city hospitals. The Indian review revealed that 60% of studies suggested antibiotics over 30% of patients' prescriptions,^21^ while Pakistan study revealed antibiotics were recommended in 9.6% of the prescriptions.^23^ In another study done in Nepal, antibiotics were provided to 9.7% patients.^22^ Though the government hospital prescribed antibiotics more than the WHO recommendation, its results was near to the Indian study. However, private hospitals prescribed not only more than the WHO recommendation but also from the mentioned south Asian countries. Irrational antibiotic prescribing has apocryphal benefits that may cause of severe reactions and hospitalization of patients. It may be responsible for the patient complications such as antibiotic resistance.^26^ For the prevention of the antibiotic resistance as well as the misuse of the antibiotics, both types of hospitals' prescriber should try to follow the WHO recommendation.

Parenteral therapy is always costlier than oral delivery system. Furthermore, irrational use such as nonsterile injections increases the risk of blood borne pathogens spreading.^4^ In this investigation, the positivity is that both government and private hospitals contained parenteral products within the range of WHO recommended value (17.70% vs. 18.35% respectively). Indian review showed that 20% percent of patients got injections,^21^ while Pakistani study demonstrated that injections were prescribed in 9.8%.^23^ In another study done in Nepal, whereas injections were given to just 0.6%.^22^ As per the comparison, both private and government hospitals contain same amount of parenteral products in their prescriptions as like as the south Asia countries.

Regarding the WHO EDL, the outcomes showed that private hospitals' prescriptions (80.42%) were contain more medicine from EDL than government (67.97%), while both did not fulfill the WHO optimal value (100%). The Indian review study showed that the percentage of drugs prescribed from the essential drug list in Government and Private hospital setting was 22.57—100% and 28.58—98%, respectively.^21^ The study of the capital of Pakistan, Islamabad, revealed that most medications (77.7%) were picked up from the EDL.^23^ Another study indicated that 65.8% of medications were administered from the EDL in Nepal.^22^ After evaluation with the south Asian countries, both types of hospitals followed the same trend on the prescribing of EDL. The EDL provides a framework for rational prescribing by including medicines that have been tested in practice, established in clinical use, and are generally less expensive than newer drugs.^27^ It is advisable for the both types of the hospital to prescribe medicine from the EDL as much as possible rather than the expensive patent drugs.

### 4.2 Various prescribing omission errors

The trend of missing age in government hospitals was not only more than the private hospitals (34.89%, vs. 9.17%) but also from the India (0%) and Nepal (0.6%), however, lower than the Pakistan (66%).^24^, ^28^, ^29^ As per the WHO, “age” is required to select the proper drug, dose, and dosage form for the right aged person, particularly for children and the elderly patients.^30^ It is advisable for the both types of hospitals especially government hospitals to take steps to reduce omission of the age.

Although private hospitals in Dhaka missed comorbidity in 28.50% of cases, this rate is lower than in India (45%), Pakistan (37.5%), and Nepal (39.2%),^24^, ^28^, ^29^ In contrast, government hospitals had rates of missed comorbidity cases similar to those in other South Asian countries.^24^, ^28^, ^29^ Notably, both types of hospitals in this study performed better than a previous Bangladeshi study, which reported a 96.75% rate of missing comorbidity information.^31^ Mentioning comorbidity is essential for proper treatment and rational use of medicines,^30^ as accurate comorbidity diagnoses are crucial for ensuring that patients receive appropriate treatment. Comorbidities provide important information to both the medication dispenser and the patient about the illness condition.^24^

Both types of hospitals omitted patients' weight, approximately to 87%, more than Pakistan (79.7%) and India (83%).^28^, ^29^ The patient's weight is needed to calculate the appropriate medication dose.^32^ When medication errors occur due to incorrect or unknown patient weights, the dose of a given drug may fluctuate significantly from the appropriate doses.^32^ It is challenging to check for any justification of the doses without weight.

Except “absence of dose,” government hospitals had a somewhat higher ratio of inscription errors than private hospitals. The “absence of dose” in both categories hospitals was around the same percentage (41.14% vs. 43.96%), higher than Nepal (32.6%) and Pakistan (9.3%).^24^, ^29^ The “absence of frequency” of government and private (10.39% vs. 6.76%, respectively) prescriptions was higher than the two studies, such as Nepal (1.1%) and Pakistan (7.3%).^24^, ^29^ However, the “absence of dosage form” was 6.25% and 3.39% in the government and private hospitals, respectively, which was lower than Pakistan (9.3%) but not than Nepal (4.5%).^24^, ^29^ The “absence of dosage duration” in the government hospitals (20.83%) is higher than the private (16.90%) and India (13%) but lower than Pakistan (46.3%).^24^, ^29^ Incorrect dose, frequency, and duration use, as well as inscription errors, might result in drug resistance, toxicological effects, and therapeutic failure.^33^ Good prescription practice is essential to minimize dose dispensing errors; physicians of both kinds of hospitals should follow protocol to benefit patients.

Private hospitals contained physician's signatures (90.33%), nearly three times greater than government (33.85%) and better than Nepal (80.8%), but not India (100%).^24^, ^28^ Another previous Bangladeshi study suggested that missing prescribing name was one of the most common prescribing errors (100%)^31^, while both private and government hospitals' of the Dhaka city were far better than the previous study. A small number of government and (5.72% vs. 0.96%) of private prescriptions missed date, approximately the same as Nepal (0.8%), but both hospitals surpass Pakistan (11%).^24^, ^29^ A signature is required to identify the doctor after prescribing, and without this, drugs cannot be given to patients, while the date is needed to preserve a record of the prescription.^24^ It is recommended for both types of hospital physicians to maintain 100% signature and date in their prescriptions.

### 4.3 Polypharmacy

Polypharmacy is one of the vital problems of the worldwide healthcare system. It is responsible for several problems such as treatment costs, adverse reactions, and drug interactions.^34^ In gender category, the number of the female patients were more prominent in both types of hospitals' polypharmacy cases (27.60% vs. 42.02%). Female patients of private hospitals may have more risk of polypharmacy, such as increasing treatment costs, risk of adverse events, and drug reactions.^34^

In age categories, prescriptions from private hospitals contained four times (19.32%) higher polypharmacy cases in the pediatric and young adults' category than the government (4.17%). Children with complex chronic diseases may be exposed to more high‐risk drugs. Moreover, polypharmacy exacerbates the high risk of drug−drug and drug‐disease interactions, which may be related to adverse drug events.^35^

Old adults are more vulnerable to adverse drug reactions due to metabolic and drug clearance changes; this risk is enhanced further by increasing the number of medications and polypharmacy.^36^ The authority of the both types of hospitals especially private hospitals should be concern about the old adults' polypharmacy incidents because as per the current study, it is two times higher than the government hospitals, moreover, old adults' are more susceptible to this.

Polypharmacy cases was predominant in private hospitals even on the single comorbidity cases. It is nearly two times greater than the government hospitals (24.15% vs. 12.5%, respectively). This statistics may indicate the presence of multiple prescribing which may account for the adverse drug reactions, drug dependency, and treatment costs.^37^

Although missing age had no association with prescription error categories, both hospitals had huge polypharmacy cases. The inclusion of age in prescriptions is required to select the appropriate medicine, dose, and dosage form for the appropriate age group, particularly for the young and the elderly.^30^ It is too difficult to calculate whether polypharmacy is in the right way or the existence of the irrationality.

The presence of the comorbidity is crucial for proper treatment and to ensure rational use of medicines and it should be mandatory.^30^ Patients can only receive proper treatment if the comorbidity is accurate besides of the proper medications.^30^ Around 20% of both prescriptions had no comorbidity but polypharmacy. Polypharmacy with a missing comorbidity appears hazardous and cannot be evaluated properly without the prescriber. It may increase the probable risk of polypharmacy‐related health problems more than other categories.

## 5 LIMITATIONS OF THE STUDY

This study, however, has several flaws. All of the samples were taken during the COVID‐19 outbreak, raising health concerns due to the need to collect prescriptions from the hospital's outdoor department. Furthermore, the study only included outdoor departments and omission errors, completely disregarding indoor departments and commission errors. Prescriptions were primarily collected directly from patients or their attendants, resulting in selective data sharing that may affect data accuracy. Additionally, the use of consecutive sampling could introduce bias into the investigation. Differences in methodology and context across countries could impact the comparability of the study results.

## 6 RECOMMENDATIONS FOR FUTURE

Both types of hospitals failed to meet WHO prescription indicators and lacked important patient and prescriber information, such as superscription, inscription, and subscription. This deficiency jeopardizes overall treatment objectives. To address these issues, here are some suggestions for hospitals' health authority:

- Avoid polypharmacy whenever feasible; when treating multiple issues, prescribe fewer medications to prevent adverse effects.
- Develop specialized software‐based prescription systems that can detect prescription gaps.
- Emphasize the need for complete physician and patient‐related information, particularly in government hospitals.
- Strongly discourage multiple and overprescribing practices.
- Improve adherence to WHO antibiotic prescription standards in both types of hospitals to address antibiotic resistance and patient noncompliance.

## 7 CONCLUSION

Prescription errors are one of the most prominent health problems worldwide. This investigation determined the degree of errors and compared Dhaka city's private and government hospitals. Both types of hospitals deviated similarly from the standard of “WHO prescribing indicator.” Both significantly omitted essential patient, physician, and drug information, such as superscriptions, subscriptions, and inscriptions, with government hospitals being more frequent in this regard. While both types of hospitals were significantly impacted by polypharmacy, this issue was more common in private hospitals. The results of this study will support the health sector in Dhaka, Bangladesh, in developing a unique prescription pattern.

## AUTHOR CONTRIBUTIONS

**Md Abdus Samad**: Conceptualization; investigation; writing—original draft; writing—review and editing; methodology; visualization; software; formal analysis; data curation; resources. **K. M. Yasif Kayes Sikdar**: Conceptualization; writing—review and editing; validation; supervision; investigation. **Ashfia Tasnim Munia**: Writing—original draft; formal analysis; software; data curation. **Farhan Tanvir Patwary**: Investigation; resources; data curation; visualization; writing—original draft; methodology; formal analysis. **Md Raihan Sarkar**: Writing—original draft; writing—review and editing; supervision; validation; formal analysis; data curation. **Md Rashidul Islam Rashed**: Writing—original draft; writing—review and editing; validation.

## CONFLICT OF INTEREST STATEMENT

The authors declare no conflict of interest.

## TRANSPARENCY STATEMENT

The lead author K. M. Yasif Kayes Sikdar affirms that this manuscript is an honest, accurate, and transparent account of the study being reported; that no important aspects of the study have been omitted; and that any discrepancies from the study as planned (and, if relevant, registered) have been explained.

## Supporting information

Supporting information.

## Data Availability

The data that support the findings of this study are available on request from the corresponding author. The data are not publicly available due to privacy or ethical restrictions.
