# Supplementary material for: Comparative analysis of prescription patterns and errors in government versus private hospitals in Dhaka: A cross‐sectional study
Source: Health Sci Rep. 2024 Aug 12;7(8):e2302. doi: 10.1002/hsr2.2302 (PMC11318024; doi:10.1002/hsr2.2302)
Supplement: Supplementary file 1 — Supporting information. [file HSR2-7-e2302-s001.docx]

**Supporting Information**

**Comparative Analysis of Prescription Patterns and Errors in Government vs. Private Hospitals in Dhaka: A Cross-Sectional Study**

Md Abdus Samad^1,2^, K. M. Yasif Kayes Sikdar^3*^, Ashfia Tasnim Munia^4^, Farhan Tanvir Patwary^5^, Md Raihan Sarkar^3^ and Md. Rashidul Islam Rashed^3^

^1^Department of Pharmacy, Faculty of Pharmacy, University of Dhaka, Dhaka-1000, Bangladesh.

^2^Department of Pharmacy, School of Pharmaceutical Sciences, State University of Bangladesh, Dhaka-1461, Bangladesh

^3^Department of Pharmaceutical Technology, Faculty of Pharmacy, University of Dhaka, Dhaka1000, Bangladesh.

^4^Institute of Statistical Research and Training, University of Dhaka, Dhaka-1000, Bangladesh.

^5^Department of Pharmacy, University of Asia Pacific, Dhaka-1205, Bangladesh.

**Email:**

Md Abdus Samadd: [abdus-2015018058@pharmacy.du.ac.bd](mailto:abdus-2015018058@pharmacy.du.ac.bd)

K. M. Yasif Kayes Sikdar: [yasif@du.ac.bd](mailto:yasif@du.ac.bd)

Ashfia Tasnim Munia: [atasnim@isrt.ac.bd](mailto:atasnim@isrt.ac.bd)

Farhan Tanvir Patwary: [tanvirfarhan@outlook.com](mailto:tanvirfarhan@outlook.com)

Md Raihan Sarkar: [raihan.rezvi@du.ac.bd](mailto:raihan.rezvi@du.ac.bd)

Md. Rashidul Islam Rashed: [rashduphr@gmail.com](mailto:rashduphr@gmail.com)

***Corresponding author**

K. M. Yasif Kayes Sikdar

Department of Pharmaceutical Technology,

Faculty of Pharmacy,

University of Dhaka, Dhaka1000.

Email: [yasif@du.ac.bd](mailto:yasif@du.ac.bd);

Phone No.: +88015152655

**Table S1**: Selected hospitals for the prescription collection through the “nth simple random sampling technique”.

| **Government hospitals** | **Frequency**  **(percentage)** | **Private hospitals** | **Frequency**  **(percentage)** |
| --- | --- | --- | --- |
| A | 25  (13.02%) | H | 19  (9.17%) |
| B | 38  (19.79%) | I | 32  (15.45%) |
| C | 21  (10.93%) | J | 24  (11.59%) |
| D | 26  (13.54%) | K | 20  (9.66%) |
| E | 18  (9.37%) | L | 26  (12.56%) |
| F | 28  (14.58%) | M | 25  (12.07%) |
| G | 35  (18.22%) | N | 33  (15.94%) |
|  |  | O | 28  (13.52%) |
| Total | 192  (100.00%) |  | 207  (100%) |

Seven different government hospitals and eight different private hospitals were selected through the “nth” random sampling technique at the time of the data collection (Table S1).
